# Apoptotic Machinery Diversity in Multiple Myeloma Molecular Subtypes

**DOI:** 10.3389/fimmu.2013.00467

**Published:** 2013-12-23

**Authors:** Patricia Gomez-Bougie, Martine Amiot

**Affiliations:** ^1^INSERM, U892, Nantes, France; ^2^Université de Nantes, Nantes, France; ^3^CNRS, UMR 6299, Nantes, France; ^4^Service d’Hématologie CHU de Nantes, Nantes, France

**Keywords:** multiple myeloma, MGUS, Bcl-2 family, *CCND1*, *MMSET, MAF*

## Abstract

Multiple myeloma (MM) is a plasma-cell (PC) malignancy that is heterogeneous in its clinical presentation and prognosis. Monoclonal gammopathy of undetermined significance (MGUS) consistently preceded development of MM. The presence of primary IgH translocations and the universal overexpression of cyclin D genes led to a molecular classification of MM patients into different disease subtypes. Since Bcl-2 family proteins determine cell fate, we analyzed a publicly available Affymetrix gene expression of 44 MGUS and 414 newly diagnosed MM patients to investigate (1) the global change of Bcl-2 family members in MM versus MGUS (2) whether the four major subtypes defined as hyperdiploid, CyclinD1, MAF, and MMSET, display specific apoptotic machineries. We showed that among the main anti-apoptotic members (Bcl-2, Bcl-x_L_, and Mcl-1), Mcl-1 up-regulation discriminated MM from MGUS, in agreement with the prominent role of Mcl-1 in PC differentiation. Surprisingly, the expression of multi-domain pro-apoptotic Bak and Bax were increased during the progression of MGUS to MM. The combined profile of Bcl-2, Bcl-x_L_, and Mcl-1 was sufficient to distinguish MM molecular groups. While specific pro-apoptotic members expression was observed for each MM subtypes, CyclinD1 subgroup, was identified as a particular entity characterized by a low expression of BH3-only (Puma, Bik, and Bad) and multi-domain pro-apoptotic members (Bax and Bak). Our analysis supports the notion that MM heterogeneity is extended to the differential expression of the Bcl-2 family content in each MM subgroup. The influence of Bcl-2 family profile in the survival of the different patient groups will be further discussed to establish the potential consequences for therapeutic interventions. Finally, the use of distinct pro-survival members in the different steps of immune responses to antigen raises also the question of whether the different Bcl-2 anti-apoptotic profile could reflect a different origin of MM cells.

## Introduction

Multiple myeloma (MM) is a plasma-cell (PC) malignancy that is heterogeneous in its clinical presentation and prognosis. Monoclonal gammopathy of undetermined significance (MGUS) consistently precedes the development of MM. The presence of primary IgH translocations and the universal overexpression of CCND (cyclinD) genes has led to a molecular classification of MM patients into different disease subtypes (1–3). The main translocations involve the immunoglobulin gene heavy chain locus on 14q32.33 with recurrent chromosome partners. These include t(11;14), t(4;14), t(14;16), and t(14;20) with an overexpression of *CCND1, MMSET, c-MAF*, and *MAFB*, respectively. Moreover, half of MM patients do not exhibit IgH translocation but present multiple trisomies involving chromosomes 3, 5, 7, 9, 11, 15, 19, and 21 and constitute the hyperdiploid subgroup of MM patients.

Impaired apoptosis is often associated with tumorigenesis and resistance to treatment. Apoptosis is controlled at multiple levels and members of the Bcl-2 family regulate the mitochondrial apoptosis pathway. They can be divided into three functional groups. The anti-apoptotic group comprises Bcl-2, Mcl-1, Bcl-xL, A1, and Bcl-w. These molecules contain four BH-2 homology domains. The pro-apoptotic multi-domain effectors, Bax and Bak, induce mitochondria damage upon activation and constitute a second group (4). Lastly, the BH3-only group, encompasses direct activators of Bax/Bak (Bid, Bim, and Puma) and sensitizers (Noxa, Bik, Bad, Hrk, and Bmf), which bind to anti-apoptotic relatives in order to induce the release of BH3 activators (5).

Individual BH3-only proteins exhibit differential affinities for their pro-survival counterparts. The activators Bim, Puma, and Bid bind all pro-survival members with high affinity, whereas BH3-only sensitizers display more selectivity. For instance, Bad binds with high affinity only to Bcl-2, Bcl-x_L_, and Bcl-w, and Noxa only to Mcl-1 and A1. Bax and Bak also differ in their interaction profile. Bak is tightly bound by Mcl-1 and Bcl-x_L_ but weakly by Bcl-2 whereas Bax seems to be neutralized by all pro-survival members (6).

The cellular content of the Bcl-2 family molecules varies among the different cell types, however it is certain that the interaction between anti-apoptotic and pro-apoptotic Bcl-2 members dictates whether a cell should die or not (4).

Since Bcl-2 family proteins determine cell fate, we analyzed a publicly available library of Affymetrix gene expression levels from 44 MGUS and 414 newly diagnosed MM patients to investigate (a) the global change of Bcl-2 family members in MM versus MGUS and (b) whether the four major MM subtypes, defined as respectively hyperdiploid (HY) or IgH translocation in 11q13 (CCND1), 16q23 (MAF), and 4p16 (MMSET), display specific apoptotic machineries.

## Results

### Analysis of Bcl-2 family expression between MGUS and MM

**Figure 1:**
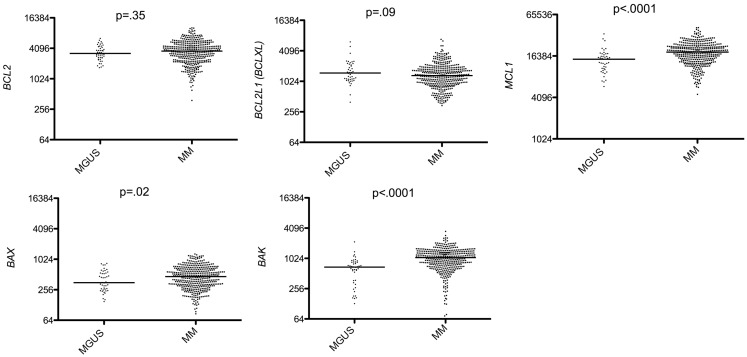
**Affymetrix data from a cohort of 44 MGUS and 414 untreated patients from the Arkansas Cancer Research Center were analyzed for *BCL2* (probe set 203685_at), *BCL2L1* (probe set 212312_at), *MCL1* (probe set 200797_s_at), *BAX* (probe set 208478_s_at), and *BAK* (probe set 203728_at) expression using the Amazonia database (http://amazonia.transcriptome.eu/)**. Median values were indicated. Statistical analyses were done using the Mann–Whitney test.

**Figure 2:**
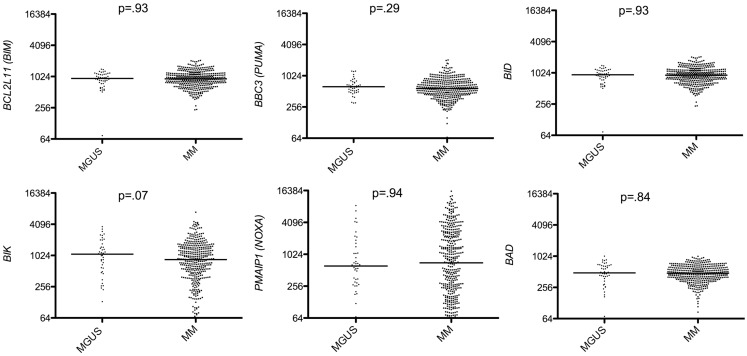
**Affymetrix data from a cohort of 44 MGUS and 414 untreated patients from the Arkansas Cancer Research Center were analyzed for *BIM* (probe set 1553088_a_at), *BBC3* (probe set 211692_s_at), *BID* (probe set 211725_s_at), *BIK* (probe set 205780_at), *PMAIP1* (probe set 204285_s_at), and *BAD* (probe set 1861_at), expression using the Amazonia database**. Median values were indicated. Statistical analyses were done using the Mann–Whitney test.

We found that among anti-apoptotic members, only Mcl-1 was significantly up-regulated in MM compared to MGUS (*p* < 0.0001 Mann–Whitney test; Figure 1) whereas *BCL2* and *BCLXL* remained unchanged or slightly decreased in MM versus MGUS (Figure 1). Of note, we excluded *BCL2A1* from the study since it is largely expressed in B cells but lost during PC differentiation. The expression of all BH3-only proteins was not modified during the progression from MGUS to MM (Figure 2). In contrast, we observed that the expression of multi-domain pro-apoptotic *BAX* and *BAK* was significantly increased in MM (*p* = 0.055 and *p* < 0.0001 respectively Mann–Whitney test; Figure 1). Altogether, the major modifications of the Bcl-2 family gene expression during the progression of MGUS to MM mainly affected *MCL1* and *BAK* gene expression (1.27 and 1.54 median fold change, respectively). These modifications between MGUS and MM should be interpreted with caution since PC populations in MGUS include both normal and malignant cells. Accordingly, it has been previously reported that normal PC in MGUS can represent up to 65% whereas in MM the percentage of normal PCs is <2% (7).

### Comparison of Bcl-2 family members in MM subgroups

**Figure 3:**
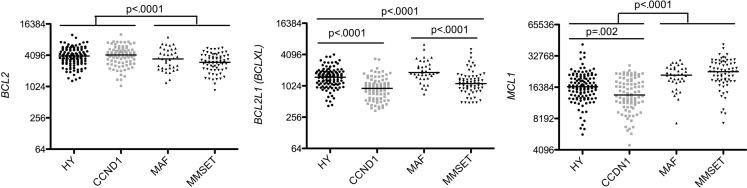
**Affymetrix data from untreated patients were analyzed for *BCL2, BCL2L1*, and *MCL1*, expression using the Amazonia database in the different molecular groups classified as follow: hyperdiploid (HY), CCND1 (CD1 + CD2), MAF, and MMSET (3)**. Probe sets are indicated in Figure 1. Median values were indicated. Statistical analyses were conducted using the Kruskall–Wallis test when more than two groups were compared and Mann–Whitney test for the comparison between two groups.

Analysis of anti-apoptotic gene expression within the four major subgroups provided evidence that the HY and CCND1 groups can be distinguished from MAF and MMSET groups by a high expression of *BCL2* and a weak expression of *MCL1* (1.3 and 0.7 median fold change, respectively), as already reported (8). Furthermore, *BCLXL* allowed to discriminate HY from CCND1 patients and also MAF from MMSET patients, since CCND1 expressed significantly less *BCLXL* than HY patients (*p* < 0.0001 Mann–Whitney test; Figure 3) and MMSET patients expressed significantly less *BCLXL* than MAF patients (*p* < 0.0001 Mann–Whitney test; Figure 3). Although *BCLXL* was heterogeneous among the four subtypes, its role in MM physiopathology remained elusive. While we have previously shown that silencing *BCLXL* did not alter the survival of myeloma cell lines (9), other studies have demonstrated that *BCLXL* played a role in chemoresistance (10). Of note, a high expression of *MCL1* was found in the worse prognosis groups (MAF and MMSET) according to the essential function of Mcl-1 in MM cell survival (9, 11, 12). On the other hand, we may question whether the lowest *MCL1* levels present in the CCDN1 group could influence its neutral outcome.

BH3-mimetic small molecules that bind to the BH3 binding sites of anti-apoptotic proteins have been developed. Among them ABT-199 is the newest one, characterized by its high potency to inhibit specifically Bcl-2 (13). In agreement with the high expression of *BCL2* in CCND1 patients, we have recently demonstrated in a small cohort of MM patients that ABT-199 sensitivity was restricted to t(11;14) patients (14). Further analysis of a larger cohort of MM patients for ABT-199 sensitivity could allow identifying HY patients able to respond to ABT-199.

**Figure 4:**
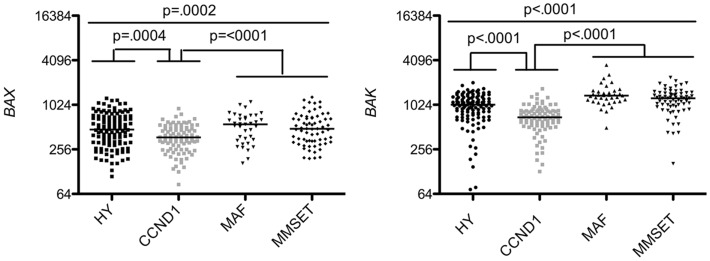
**Affymetrix data from untreated patients were analyzed for *BAX* and *BAK* expression using the Amazonia database in the different molecular groups classified as above in Figure 3**. Probe sets are indicated in Figure 1. Median values were indicated. Statistical analyses were conducted as in Figure 3.

**Figure 5:**
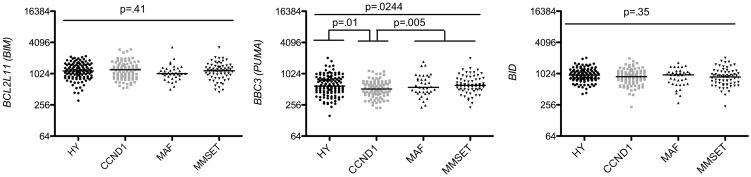
**Affymetrix data from untreated patients were analyzed for *BIM, BBC3*, and *BID*, expression using the Amazonia database in the HY, CCND1, MAF, and MMSET molecular groups**. Probe sets are indicated in Figure 2. Median values were indicated. Statistical analyses were conducted as in Figure 3.

Strikingly, analysis of multi-domain pro-apoptotic members showed that the worse prognosis groups MAF and MMSET displayed higher levels of *BAX* and *BAK* in contrast to the CCDN1 group, which expressed the lowest levels of both effectors (1.38 and 1.85 median fold change, respectively) (Figure 4). BH3-only activators (*BIM, PUMA, BID*) were constantly expressed in the four subtypes, suggesting that independently of the subtype, MM cells are primed for death, as already reported (15, 16) (Figure 5). Of note, Puma expression was weaker exclusively in CCDN1 group compared to the other subgroups (0.82 median fold change). The promiscuous binding of Bim and Puma to main pro-survival members, associated with the fact that knockout mice for them do not present a particular phenotype, suggest a complementary role for these two members (17). However, based on the constant endogenous expression of *BIM* and *PUMA* found in the four MM subgroups, it would be pertinent to address the question whether they have complementary roles or not in this pathology.

**Figure 6:**
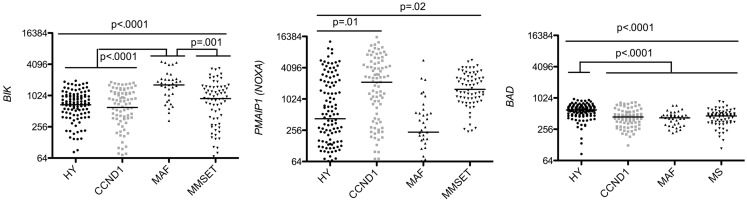
**Affymetrix data from untreated patients were analyzed for *BIK, PMAIP1*, and *BAD* expression using the Amazonia database in the HY, CCND1, MAF, and MMSET molecular groups**. Probe sets are indicated in Figure 2. Median values were indicated. Statistical analyses were conducted as in Figure 3.

In contrast to BH3-only activators, sensitizers (*BIK, NOXA, BAD*) were heterogeneously expressed in the different MM subtypes. However, each MM subgroup highly expressed at least one sensitizer (Figure 6). These results are consistent with the fact that sensitizer BH3-only proteins may have overlapping functions (18). In this respect, it was previously shown that knockout mice for either Bik or Noxa proteins do not develop spontaneous tumors (17, 19). Interestingly, we found that few patients in some subgroups lacked Bik, according to our previous finding showing that some MM cell lines do not express Bik at the protein level (20). Deletions and epigenetic alterations have been shown to contribute to the lack of Bik expression (21, 22). TEF, a PARbZIP transcription factor, was identified as a direct activator of BIK promoter (21). We have shown in MM cell lines, that Bik was expressed only in the presence of TEF mRNA (20). However, despite TEF expression, some cell lines did not express Bik. Altogether, these results suggest that lack of Bik might be the result of either an epigenetic alteration or a deletion, as frequently described in other cancers (22).

In addition, *NOXA* allowed discriminating CCND1 from HY patients (5 median fold change) and also MMSET from MAF patients (6.68 median fold change) (Figure 6). Although the difference of *NOXA* expression was impressive among the four subtypes, its role in MM physiopathology remained to be determined.

Hyperdiploid patients expressed significantly higher levels of *BAD* than all other subgroups (1.35 median fold change) (Kruskall–Wallis *p* < 0.0001), which may be explained by the localization of *BAD* on chromosome 11q1 (Figure 6). Indeed, chromosome 11 trisomy is one the most frequent anomaly in the HY subgroup (23).

## Concluding Remarks

Noteworthy, our analysis demonstrated that the combined profile of the three anti-apoptotic molecules (Bcl-2, Bclx-_L_, and Mcl-1) was sufficient to discriminate the different MM molecular groups. The CCDN1 subgroup was identified as a particular entity, characterized by a *BCL2^high^* *MCL1^low^* and a low expression of pro-apoptotic effectors and BH3-only *(PUMA, BIK*, and *BAD*) with the exception of high expression of *NOXA*. Since Noxa interacts only with Mcl-1, which is weak in this subtype, we can hypothesize that the anti-apoptotic function of Mcl-1 is totally neutralized by Noxa and that this subtype relies mainly on Bcl-2 for survival. Altogether, the apoptotic machinery of this myeloma subtype is very different from that of other subtypes, suggesting that specific therapeutic approaches should be investigated to target CCDN1 patients. We can also hypothesize that the specificity of this subgroup may reflect a specific origin of malignant PC immortalization. This is also supported by the fact that patients harboring a t(11;14) have a high prevalence of IgM isotype and represent a distinct biological and clinical subgroup (24). Furthermore, recurrent translocations also correlated with particular features (25). Indeed, t(11:14) translocations exhibited a mature lymphoplasmocytoid morphology with a higher incidence of non-secretory MM. In contrast, t(4;14) MM subtype revealed a morphology of immature plasma cells with a significant amount of plasmablasts (25, 26). The HY subgroup shares some similarities with CCND1 in the expression pattern of Bcl-2 family members, particularly high *BCl2* and low *MCL1* levels. However, HY patients expressed higher levels of pro-apoptotic members (*BAX, BAK*, and *BAD*) than those of the CCND1 subgroup (Figures 5 and 6). This could favor the apoptotic response to chemotherapy and therefore explain in part the better outcome of these patients. In contrast, MAF and MMSET subgroups differ from CCDN1 and HY in the expression of anti-apoptotic members and were characterized by low *BCL2* and high *MCL1* levels. Surprisingly, MAF and MMSET subgroups expressed high level of effectors, particularly Bak, suggesting their ability to trigger an effective drug response. It will be intriguing to define whether the high expression of effectors in MAF and MMSET subgroups may be related to the fact that bortezomib-containing regimens could overcome the poor prognosis associated with t(4;14) (27). Paradoxically, these subgroups have the poorest outcome, highlighting a potential role of Mcl-1 in chemoresistance. A potential and interesting approach to target efficiently MAF and MMSET patients would be to use pharmacological inhibitors of Mcl-1, which are currently under development (28).

To summarize, our analysis supports the notion that MM heterogeneity extends to the composition of the Bcl-2 family content in each MM subgroup, which should be taken into account for therapeutic intervention in the new approach of personalized therapies. Finally, a better knowledge of Bcl-2 expression patterns may be relevant to address the origin of malignant PC.

## Conflict of Interest Statement

The authors declare that the research was conducted in the absence of any commercial or financial relationships that could be construed as a potential conflict of interest.
